# Evaluation of the Effect of Escitalopram on Alveolar Bone Levels and Periodontal Parameters: A Case–Control Study

**DOI:** 10.1155/ijod/8637908

**Published:** 2025-07-24

**Authors:** Ayman Ahmed, Ehab Azab

**Affiliations:** ^1^Department of Oral Medicine and Periodontology, Faculty of Dentistry, Cairo University, Cairo, Egypt; ^2^Department of Basic and Clinical Oral Sciences, Faculty of Dental Medicine, Umm Al-Qura University, Makkah, Saudi Arabia

**Keywords:** bone levels, escitalopram, periodontal parameters

## Abstract

**Objectives:** The purpose of the present study was to analyze the effect of escitalopram, a selective serotonin reuptake inhibitor, clinically and radiographically on bone levels and periodontal parameters.

**Materials and methods:** A sample of 100 patients with clinical depression was assessed for clinical periodontal parameters including plaque index (PI), gingival index (GI), probing depth (PD), and clinical attachment loss (CAL) and radiographic marginal bone loss. A total of 50 patients were administered 10 mg escitalopram daily for 3–6 months, while the other 50 patients did not receive it. *p*-Values < 0.05 with a 95% confidence interval were considered statistically significant.

**Results:** No statistically significant difference in the PI was found among the study groups (*p* > 0.05). Conversely, a significantly lower GI, PD, and CAL were found in the escitalopram users group (*p* < 0.001). Moreover, mesial and distal radiographic marginal bone loss were significantly lower in the escitalopram users group than in the nonescitalopram users group (control) (*p* < 0.001).

**Conclusion:** The present study indicates that escitalopram intake in patients with clinical depression is associated with lower periodontal parameter scores and decreased marginal bone loss.

## 1. Introduction

Periodontitis is a complex disease involving several risk factors that modify the host's response to bacterial plaque, which activates several cellular and biochemical events and ends with periodontal tissue destruction and alveolar bone loss [1]. Previous studies have shown that psychological diseases, including depression, like other systemic diseases, could affect periodontium through various behavioral and psychological mechanisms. The type of medication used for treatment could also impact periodontal tissue [2, 3].

Depression is a mental state of curtailed mood and deprivation of interest in activities or pleasure for long periods. According to the World Health Organization, more than 280 million people, about 3.8% of the global population, suffer from depression [4]. A recent meta-analysis reported a significant association between periodontitis and depression [5]. Antidepressants are classified according to their pharmacological action into five major groups: selective serotonin reuptake inhibitors (SSRIs), atypical antidepressants, tricyclic antidepressants, serotonin-norepinephrine reuptake inhibitors, and monoamine oxidase inhibitors.

SSRIs prevent the reabsorption and deactivation of serotonin in neurons, therefore elevating serotonin levels in the brain. Few studies have centered on the relationship between the use of fluoxetine, a widely used selective serotonin reuptake inhibitor (SSRI) antidepressant, and periodontitis, and the findings have been inconclusive and contradictory. Branco-de-Almeida et al. [6] showed that fluoxetine intake diminishes alveolar bone destruction in rats with ligature-induced periodontitis. Similarly, a systematic review of experimental studies in rats established that fluoxetine had a significant positive impact on bone levels [7]. Bhatia et al. [8] observed that periodontitis patients undergoing fluoxetine therapy had better clinical periodontal parameters than nonusers. On the other hand, a more recent case–control study reported higher periodontal indices in fluoxetine users than in nonusers [9].

Escitalopram, an SSRI commonly used to treat major depressive disorder and generalized anxiety disorder, offers a range of potential benefits. It is sometimes prescribed off-label for conditions, such as bulimia nervosa, premenstrual dysphoric disorder, post-traumatic stress disorder, and obsessive–compulsive disorder. In 2020, escitalopram was the 15^th^ most widely prescribed antidepressant medication in the United States, with over 30 million prescriptions. While fluoxetine and escitalopram boost serotonin levels in the brain, they have dissimilar chemical structures and may affect the body and brain differently. A recent important systematic review comparing 21 commonly used antidepressants concluded that escitalopram was the most effective, while fluoxetine was the least [10]. Moreover, insomnia was found to be a more common side effect with fluoxetine than with escitalopram, while escitalopram was identified as the best tolerated, safest antidepressant for the short-term treatment of depressive disorders [10]. These findings have led many psychiatrists to favor escitalopram over fluoxetine in their prescriptions. However, to the best of our knowledge, the effect of escitalopram on the periodontium has not yet been studied. Thus, the present study aimed to evaluate the effect of escitalopram clinically and radiographically on bone levels and various periodontal parameters.

## 2. Materials and Methods

### 2.1. Ethical Approval

The Research Ethics Committee of the Faculty of Dentistry, Cairo University reviewed and approved the study (Approval Number: 2023/36-11).

### 2.2. Study Design and Setting

The current study is a case–control conducted at the Department of Periodontology, Faculty of Dentistry, Cairo University from November 2023 to February 2024.

### 2.3. Study Population

Inclusion criteria:

1. Patients diagnosed by a psychiatrist as having depression based on the International Classification of Diseases ICD-10 symptom rating (ISR) [11].
2. Adult patients ranging from 30 to 60 years.
3. Presence of ≥20 natural teeth (excluding third molars).
4. Self-reported detailed medical history of free systemic diseases other than depression.

Exclusion criteria:

1. Current or past smokers and tobacco chewers.
2. Pregnant and lactating women.
3. Patients who received surgical or nonsurgical periodontal therapy in the previous 6 months.
4. Patients on antibiotics, steroids, nonsteroidal anti-inflammatory drugs, bisphosphonates, vitamins, or any nutritional supplements for the previous 6 months.

All participants were recruited from the psychiatric department of the Faculty of Medicine at Cairo University after signing an enlightened consent form. The sample size was determined to be 100 participants based on previous research [12] and were categorized into two groups:

Group I (control group): Comprising 50 individuals diagnosed by a psychiatrist as having depression and not having received antidepressants.

Group II: Comprising 50 individuals diagnosed by a psychiatrist as having depression and taking escitalopram 10 mg/day for 3–6 months.

### 2.4. Data Collection

Demographic data, including age, gender, educational level, and oral hygiene measures, were obtained from each participant, as well as a full-mouth plaque index (PI) [13], gingival index (GI) [14], probing depth (PD), and clinical attachment loss (CAL). Measurements were taken in millimeters using a Williams periodontal probe and approximated to the next mm. Full-mouth digital bitewing radiographs were conducted to apprize the marginal bone loss (MBL) measured as linear distances from 2 mm below the cementoenamel junction (CEJ) to the alveolar crest. One examiner performed the periodontal examination to preclude inter-examiner variability.

### 2.5. Statistical Analysis

Data were gathered, tabulated, and then analyzed using the statistical package for social sciences (SPSS) version 24 software. The Shapiro–Wilk test was used to assess data normality, while comparisons were evaluated using the Mann–Whitney *U* test. *p*-Values < 0.05 with a 95% confidence interval were considered statistically significant.

## 3. Results

A clinical and radiographic evaluation of 100 participants diagnosed by a psychiatrist as having depression was undertaken. All participants had mild depression based on the ISR. Of these, 50 individuals were prescribed escitalopram 10 mg/day per medical records for 3–6 months, and 50 individuals who had not yet, received escitalopram or any other antidepressant were used as controls. Of the 50 individuals with depression and nonescitalopram users, 24 (48%) were male, and 26 (52%) were female. Of the 50 escitalopram users, 23 (46%) were male, and 27 (54%) were female. The mean ages of the escitalopram users and controls were 47.3 ± 2.4 and 44.9 ± 3.8 years, respectively. A total of 96% of escitalopram users and 92% of controls had a university-level education, while 4% of escitalopram users and 8% of controls had a secondary school-level education. Twice daily tooth brushing was reported by 11 escitalopram users (22%) and nine controls (18%). Once-daily tooth brushing was reported by 17 escitalopram users (34%) and 14 controls (28%), while less frequent tooth brushing was reported by 22 escitalopram users (44%) and 27 controls (54%). Nine escitalopram users (18%) and seven controls (14%) reported once-daily flossing. No significant differences were noted between the escitalopram users and control groups (*p* > 0.05) (Table 1).

Table 2 shows the periodontal and radiographic status of the nonescitalopram users (controls) and escitalopram users. No statistically significant difference was found for the PI among the study groups (*p* > 0.05). Conversely, a significantly lower GI, PD, and CAL were found in the escitalopram users group (*p* < 0.001). Moreover, mesial and distal radiographic MBL were significantly lower in the escitalopram users group than in nonescitalopram users (controls) (*p* < 0.001).

## 4. Discussion

The present study was performed to clinically and radiographically evaluate the effect of escitalopram on periodontal status by comparing different periodontal parameters and marginal bone levels in patients with depression receiving escitalopram for 3–6 months with congruent diagnostic coordinated individuals who had not yet received antidepressants for the treatment of depression. It found no significant differences in average age, gender, education level, oral hygiene measures (including toothbrushing and flossing frequency), and the PI between escitalopram users and nonescitalopram users in the control group.

There is increasing evidence that depression is associated with an upregulated inflammatory response, as expressed by the increased production of pro-inflammatory cytokines. Interestingly, this pro-inflammatory hypothesis, which asserts that pro-inflammatory cytokines as delegates induce depressive symptoms, has evolved from sound scientific research. Periodontal pathogen endotoxins are associated with an increase in pro-inflammatory cytokine levels, which may increase vulnerability to depression. Studies have shown that the experimental administration of tumor necrosis factor alpha (TNF-α), interleukin-1 beta (IL-1β), and IL-6 results in a depressive effect, including anxiety, drowsiness, and fatigue, in humans. Pro-inflammatory cytokines were found to activate the hypothalamus–pituitary–adrenal axis, causing an increase in cortisol levels, decreasing antioxidative mechanisms, harming neuroplasticity, affecting the tryptophan metabolism, and decreasing serotonin synthesis [15, 16]. Furthermore, a recent meta-analysis and systematic review demonstrated the vital role of inflammation in the pathogenesis, severity, and treatment outcomes of depression [17].

The GI is an inflammatory parameter that can be used to appraise the quantity and severity of gingival inflammation and is regarded as a clinical sign of inflammatory periodontal disease. Our finding of significantly lower GI scores in the escitalopram users group than in the non-escitalopram users control group suggests that patients with depression receiving escitalopram are better protected against periodontal inflammation. Moreover, the results of the present study demonstrated that the PD and CAL, as well as the mesial and distal radiographic MBL, were significantly lower among escitalopram users than among the nonescitalopram users control group. This finding could reflect the impact of a modifying factor that decreases the severity and extent of periodontal disease observed in participants taking escitalopram. The nonsignificant difference in PI scores in the study indicates that controlling depression in escitalopram users could surpass clinical parameters due to a greater inclination to oral hygiene.

The improved periodontal parameters in patients receiving escitalopram may be attributed to the anti-inflammatory and immunomodulatory effects of escitalopram, which can decrease the production of inflammatory mediators implicated in periodontal destruction. In a lipopolysaccharide-induced inflammatory model of depression, escitalopram demonstrated antidepressant and anti-inflammatory effects by significantly reducing the lipopolysaccharide-induced ascend in serum TNF-α in rats [18]. Likewise, escitalopram was proven to inhibit the production of Th2 cytokines, such as IL-6 [19]. Moreover, a previous systematic review and meta-analysis reported that antidepressant treatment significantly reduced peripheral levels of monocyte chemoattractant protein 1, IL-8, TNF-α, IL-1β, and IL-6, which are elevated in depression, and granulocyte macrophage colony-stimulating factor that upgrades the activation and survival of macrophages, dendritic cells, and neutrophils [17]. The escitalopram-induced reduction in proinflammatory cytokines implicated in bone resorption could account for the lower bone loss shown in our results. A critical review of the action mechanism of SSRIs showed that SSRIs decrease cytokine production and peripheral immune cell proliferation [20]. Escitalopram-induced elevated serotonin levels have been found to protect the periodontium from inflammation and prevent oxidative damage [21]. In addition, escitalopram has been shown to act as an agonist, activating the sigma-1 receptor protein in the endoplasmic reticulum, which leads to several beneficial effects, including anti-inflammatory and antioxidant effects [22]. This activation reduces inflammation by suppressing the production of pro-inflammatory cytokines, such as TNF-α, while promoting the production of anti-inflammatory cytokines like IL-10. Furthermore, it enhances antioxidant effects by increasing the levels of endogenous antioxidants, like glutathione (GSH) and heme oxygenase-1 (HO-1), and decreasing oxidative stress via minimizing the formation of reactive oxygen species (ROS).

Many studies have considered the effects of antidepressants on the immune system. Medina-Rodriguez et al. [23] reported higher levels of IL-6 and TNF-α in patients treated with SSRIs. However, a meta-analysis of 35 clinical studies over more than 20 years showed that patients taking antidepressants had significantly reduced levels of TNF-α and IL-6 in their serum [24]. Another meta-analysis of the effect of antidepressant treatment on inflammatory cytokine serum levels indicated that IL-1β and TNF-α levels were significantly decreased after the use of SSRIs antidepressants in patients [25]. Similarly, a recent meta-analysis based on 32 studies measuring the levels of cytokines before and during antidepressant treatment noted significant reductions of IL-6 and IL-1β after antidepressant treatment [26]. The results of previous studies have varied because the posttreatment cytokine levels differ depending on chronicity, recurrence of depressive episodes, and severity and may vary between drug molecules.

Our results aligned with those identified in a systematic review of experimental studies in rats evaluating three antidepressants (venlafaxine, tianeptine, and fluoxetine), which showed that these medications had a significant positive influence on alveolar bone loss [4]. Similarly, fluoxetine had documented suppressive effects on periodontal disease severity and inflammatory response in a rat ligature-induced periodontitis model [6, 27, 28] and in patients with periodontitis who had clinical depression [8]. However, a recently published paper demonstrated that patients taking antidepressants had greater odds of acquiring generalized moderate periodontitis, which contradicted our results [29]. Periodontitis in that study was defined based on PD, and no data concerning CAL and bone loss were provided. In contrast to our research, Zhou et al. [30 ] investigated the effect of SSRIs on bone mineral density in the lumbar spine, total hip, and femoral neck. Their findings demonstrated that SSRIs lower the bone mineral density of the lumbar spine, particularly in older individuals, with no significant impact on the density of other bones [30]. However, our study focused on the effect of SSRIs on alveolar bone levels and included a wide range of ages.

Another study reported higher GI, PD, and CAL in SSRI users than in the non-SSRI users control group [9], although, the SSRIs used in this study were fluoxetine and venlafaxine and were taken by participants with moderate to severe depression whose education level was not assessed. In the present study, all participants had mild depression, and 96% of escitalopram users had university-level education. The suspected lower education level and the more severe levels of depression could have compelled the participants to neglect routine oral hygiene measures and compliance with the intake of SSRIs.

The present study has some limitations in that it is a single-center study, and the parameters were registered at a given time. A longitudinal study that follows groups over time is recommended. Additionally, oral microbiota may play a role in mental health conditions, such as depression. However, this study did not investigate the composition of the oral microbiome at baseline and after treatment and future research is recommended to explore the effects of escitalopram on oral microbiota.

## 5. Conclusion

The present study indicates that escitalopram intake in patients with clinical depression is associated with lower periodontal parameters scores and decreased marginal bone loss. However, these preliminary findings could not be a solid base to use escitalopram as a therapeutic agent to treat inflammatory periodontal diseases till confirmed by larger sample, well-designed, multicenter prospective cohort studies.

## Figures and Tables

**Table 1:** Demographic data.

Variables	Nonescitalopram users (controls), (*N* = 50)	Escitalopram users (*N* = 50)	Total (*N* = 100)	*p*-Value
Gender				
Male	24 (48%)	23 (46%)	47 (47%)	0.58
Female	26 (52%)	27 (54%)	53 (53%)	—
Age (years) mean ± SD	44.9 ± 3.8	47.3 ± 2.4	—	0.72
Highest education level				
Primary	N/A	N/A	N/A	—
Secondary	4 (8%)	2 (4%)	6 (6%)	0.71
University	46 (92%)	48 (96%)	94 (94%)	—
Tooth brushing				
Twice daily	9 (18%)	11 (22%)	20 (20%)	0.69
Once daily	14 (28%)	17 (34%)	31 (31%)	—
Less frequently	27 (54%)	22 (44%)	49 (49%)	—
Once daily flossing	7 (14%)	9 (18%)	16 (16%)	0.61

**Table 2:** Periodontal and radiographic status.

Variables	Nonescitalopram users (controls)	Escitalopram users	*p*-Value
Plaque index (PI)	1.74 ± 0.81	1.63 ± 0.72	0.08
Gingival index (GI)	1.9 ± 0.54	1.14 ± 0.38	<0.001^*∗*^
Probing depth (PD) (mm)	4.79 ± 0.38	3.88 ± 0.34	<0.001^*∗*^
Clinical attachment loss (CAL) (mm)	3.08 ± 0.57	2.32 ± 0.40	<0.001^*∗*^
Mesial marginal bone loss (mm)	3.17 ± 0.24	2.54 ± 0.18	<0.001^*∗*^
Distal marginal bone loss (mm)	3.21 ± 016	2.59 ± 0.26	<0.001^*∗*^

*⁣* ^*∗*^ *p*-Value < 0.05 indicates statistical significance.

## Data Availability

The data that support the findings of this study are available from the corresponding author upon reasonable request.
